# Prostate cancer tends to metastasize in the bone-mimicking microenvironment via activating NF-κB signaling

**DOI:** 10.7555/JBR.32.20180035

**Published:** 2018-06-26

**Authors:** Haibo Tong, Chunlin Zou, Siyuan Qin, Jie Meng, Evan T. Keller, Jian Zhang, Yi Lu

**Affiliations:** 1. Key Laboratory of Longevity and Aging-related Diseases, Guangxi Medical University, Ministry of Education, Nanning, Guangxi 530021, China; 2. Southern University of Science and Technology School of Medicine, Shenzhen, Guangdong 518055, China; 3. Department of Pathology and Internal Medicine, University of Michigan, Ann Arbor, MI 48109, USA.

**Keywords:** prostate cancer, metastasis, NF-κB, Bay 11-7082, EMT

## Abstract

Prostate cancer preferentially metastasizes to the bone. However, the underlying molecular mechanisms are still unclear. To explore the effects of a bone-mimicking microenvironment on PC3 prostate cancer cell growth and metastasis, we used osteoblast differentiation medium (ODM; minimal essential medium alpha supplemented with *L*-ascorbic acid) to mimic the bone microenvironment. PC3 cells grown in ODM underwent epithelial-mesenchymal transition and showed enhanced colony formation, migration, and invasion abilities compared to the cells grown in normal medium. PC3 cells grown in ODM showed enhanced metastasis when injected in mice. A screening of signaling pathways related to invasion and metastasis revealed that the NF-κB pathway was activated, which could be reversed by Bay 11-7082, a NF-κB pathway inhibitor. These results indicate that the cells in different culture conditions manifested significantly different biological behaviors and the NF-κB pathway is a potential therapeutic target for prostate cancer bone metastasis.

## Introduction

Prostate cancer (PCa), a common malignancy in men of western countries^[1]^, is the second leading cause of cancer-related deaths in men^[2]^. In China, the incidence of PCa has grown rapidly in the recent decades. Androgen deprivation therapy and chemotherapy are regarded as effective strategies in treating advanced PCa patients^[3]^. PCa often initially responds to chemotherapy, but gradually acquires drug-resistance, leading to bone metastasis and eventually, treatment failure^[4–5]^. The bone is the common site for metastasis in PCa. However, the molecular mechanism underlying this preferential metastasis remains unclear. The specific biomarkers that predict bone metastasis have not been identified. Therefore, it is imperative to elucidate the mechanisms of bone metastasis in order to develop novel therapeutic methods for patients with metastatic, chemo-resistant, and hormone-refractory prostate carcinoma.

Transcription factors of the nuclear factor κB (NF-κB)/Rel family promote the expression of inflammatory cytokines and apoptosis-inhibitory proteins^[6]^. Several genes that mediate tumorigenesis and metastasis are regulated by the NF-κB signal pathway^[7]^. In mammals, the NF-κB family members RelA, c-Rel, RelB, NF-κB1 (p105/p50), and NF-κB2 (p100/p52) form a homo- or heterodimeric complex^[8–10]^. NF-κB is sequestered in the cytoplasm by the IκBα inhibitory protein. In the conventional activation way, a highly diverse group of extracellular signals, including carcinogens, inflammatory cytokines, and chemokines, can mediate the phosphorylation of IκBα at serine 32 and 36, inducing proteolytic IκBα degradation and NF-κB activation. The activated NF-κB then translocates to the nucleus where it regulates target gene expression^[11–13]^. Bay 11-7082 has been shown to inhibit IκBα phosphorylation and prevent NF-κB from translocating to the nucleus to activate the target genes^[14]^. Emerging evidence suggests that the NF-κB pathway is involved in cell cycle regulation, cell proliferation, apoptosis^[15–16]^, epithelial-mesenchymal transition (EMT)^[17]^, migration, invasion^[18]^, and the development of drug resistance in human cancers.

Suitable tumor microenvironment can promote tumor cell growth and then lead to tumor progression and metastasis^[19]^. Therefore, bone microenvironment is key to PCa skeletal metastasis^[20]^. We hypothesized that some soluble factors in the bone microenvironment that promote osteoblast or osteoclast differentiation may contribute to PCa cell growth in the bone. In this study, we grew PCa cells in osteoblast differentiation medium to mimic the bone microenvironment and observed their characteristics in comparison to PCa cells grown in normal medium *in vitro.* Further, we observed the characters of PCa cells in a mouse model *in vivo*.

## Materials and methods

### Cell culture and reagents

Human PCa PC3 cells were obtained from the American Type Culture Collection (ATCC, Rockville, MD, USA) and maintained in RPMI-1640 medium (1640) with 10% fetal bovine serum (FBS) and 1% penicillin/streptomycin (Invitrogen, Carlsbad, CA, USA). Osteoblast differentiation medium (ODM) consisting of minimal essential medium alpha (α-MEM) supplemented with L-ascorbic acid (AA, 50 μg/mL) (Sigma, St. Louis, MO, USA) as well as 10% FBS and 1% penicillin/streptomycin was used to mimic the bone microenvironment. PC3 cells were cultured in ODM for two weeks prior to the experiments, and then used as inducible cells.

### Inhibition of NF-κB signaling

Bay 11-7082, an irreversible inhibitor of IKKα and phosphorylation of cytokine-inducible IκBα, was purchased from Sigma. The compound was dissolved in dimethylsulfoxide (DMSO, Sigma). PC3 tumor cells were grown in ODM or 1640. When the cells were nearly confluent (>80%), the inhibitor (5 μmol/L) or an equivalent volume of DMSO as vehicle control was added to the medium for 2 hours. After the treatments, the cells were washed twice with cold phosphate-buffered saline (PBS) and harvested in lysis buffer to extract proteins.

### Colony formation assays

Cells were seeded in 6-well plates at a density of 400 cells/well and cultured for approximately 10 days at 37°C in a 5% CO_2_ incubator. Then, the cultures were washed with 1×PBS, fixed with 4% formaldehyde, and stained with crystal violet (Beyotime, Shanghai, China) for 20 minutes. For inhibition assays, the cells were treated with Bay 11-7082 (1.25, 2.50, and 5.00 μmol/L) or DMSO for 10 days and the medium was replaced with fresh medium containing fresh Bay 11-7082 every 5 days. Colonies containing 50 or more cells were counted with Image J (National Institutes of Health, Bethesda, MD, USA). The colony was calculated as: colony formation rate (%) = number of colonies/number of plated cells ×100%. Three experiments were performed independently.

### Wound-healing assay

To evaluate the migration ability of PC3 cells in 1640 and ODM, wound healing assays were performed, as previously described^[21]^. The cells were seeded in 6-well plates at 1×10^6^ cells/well and cultured overnight until monolayers were entirely confluent. Multiple scratch wounds were generated in the monolayers using a 200-μL pipette tip after being treated with 20 μg/mL of mitomycin C (Roche Diagnostics GmbH, Mannheim, Germany) for 2 hours at 37 °C in the presence of 5% CO_2_. The cells were then washed with 1× PBS to remove floating cells and photographed using Olympus Cell microscope system (Olympus, Tokyo, Japan) at indicated time points (0, 8, 16, and 24 hours). The migration area was measured and analyzed with Image-Pro Plus software (Media Cybernetics, Silver Spring, MD, USA). The effect of Bay 11-7082 treatment on tumor cell migration was also evaluated by wound-healing assay. Three experiments were performed independently.

### Transwell assays

Invasion was evaluated by Transwell (Corning, NY, USA) assays, as previously described^[21]^. PC3 cells were collected and resuspended in serum-free cell culture medium with 1.5 ×10^5^ cells transferred into the upper compartments. The lower chamber contained medium containing 15% FBS as a chemoattractant. After a 15-hour incubation, the non-migrating or non-invading cells in the upper chamber were gently removed using a cotton swab. Invaded cells were fixed with formaldehyde, stained with crystal violet, air dried, photographed, and counted using Image J.

### Western blotting assays

Cell lysates were collected using standard procedures^[22]^. Protein concentrations were determined by BCA assay (Pierce, Rockville, IL, USA). Proteins were separated by 10% sodium dodecyl sulfate polyacrylamide gel electrophoresis and electroblotted onto polyvinylidene fluoride membranes. The membranes were blocked in 5% non-fat milk for 1.5 hours at room temperature with shaking, then incubated overnight in the presence of primary antibodies against MEK1/2 (#4694, 1:1,000), p44/42 MAPK (#4695, 1:1,000), phospho-MEK1/2 (#9127, 1:1,000), phospho-p44/42 MAPK (Erk1/2) (#4370, 1:1,000), phospho-NF-κB p65 (#3033, 1:1,000), phospho-IκBα (#2859, 1:1,000), NF-κB p65 (#8242, 1:1,000), β-catenin (#8480, 1:1,000), E-cadherin (#3195, 1:1,000), N-cadherin (#13116, 1:1,000), Snail (#3879, 1:1,000), Slug (#9585, 1:1,000), and GAPDH (#5174, 1:10,000) (all from Cell Signaling Technology, MA, USA). Blots were washed and incubated for 1 hour with the corresponding horseradish peroxidase-conjugated secondary antibodies following the manufacturer’s instructions. Protein bands were visualized using chemiluminescence reagent (Amersham Biosciences, Piscataway, NJ, USA). GAPDH was used as a loading control. Images were scanned using MiniChemi™ 610 Plus (Sagecreation Service for Life Science, Beijing, China), followed by densitometry with Image J. All experiments were repeated at least three times.

### Animals

The animal experimental protocol was approved by the Institutional Animal Ethics Committee (IAEC), Guangxi Medical University. Animal study was carried out in strict accordance with the established institutional guidelines on the use of experimental animals.

Nude mice [male, (15.0±2.0) g] of 6–7 weeks old were purchased from Beijing HFK Bio-technology Corporation, China. All mice were housed under specific pathogen-free conditions in accordance with National Institutes of Health (NIH) guidelines. To evaluate their effect on tumor growth and metastasis in a bone-mimicking microenvironment *in vivo*, inducible PC3 cells (2 × 10^5^ cells/mouse) were injected into the left ventricle of nude mice (*n* = 13 mice/group). Body weight and tumor volume were recorded twice a week and tumor growth was monitored weekly using the Bruker *in-vivo* multispectral (MS) FX PRO imaging system (Bruker, Billerica, MA, USA). At the end of animal test, the mice were sacrificed with the major organs and lumbar vertebrae harvested for histological analysis.

### Hematoxylin and eosin (H&E) staining

HE staining was conducted based on the previous study^[23]^. Briefly, paraffin-embedded tissues were cut into 4-μm longitudinal sections. After deparaffinization and rehydration, the sections were stained with hematoxylin solution for 10 minutes followed by 1% acid ethanol and rinsed in distilled water. Then, the sections were stained with eosin solution for 2 minutes, followed by dehydration with graded alcohol and clearing in xylene. The slides were examined and photographed under a microscope to observe the histological changes in lung and liver tissues.

### Statistical analysis

Data were presented as mean±standard deviation (SD) unless indicated otherwise. Statistical analyses were performed using GraphPad Prism software (GraphPad Software Inc., La Jolla, CA, USA). Student’s *t*-tests were used to compare means of data in Western blotting analysis, cell migration, invasion, and colony formation assays, and *in-vivo* experiments. Multiple means were compared using one-way analysis of variance (ANOVA) followed by Tukey’s post-hoc test. *P*<0.05 was considered statistically significant. All experiments were conducted at least three times independently.

## Results

### PC3 cells underwent epithelial-mesenchymal transition ***in vitro*** in bone-mimicking microenvironment

**Fig.1:**
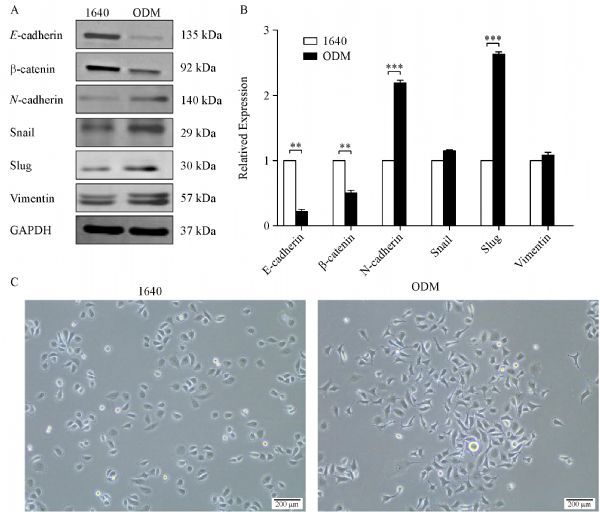
Prostate cancer PC3 cells undergo epithelial-mesenchymal transition (EMT) in ODM.

EMT is a cell remolding process key to embryonic development, as well as tissue and organ formation. It is also the initial step of tumor metastasis. To examine the functional changes of PC3 cells in a bone-mimicking microenvironment, we cultured the cells in ODM and 1640, and then evaluated EMT hallmarks. PC3 cells grown in ODM showed significantly lower expression levels of E-cadherin and β-catenin, whereas the expression levels of vimentin, slug, and snail were apparently higher, compared with those grown in 1640. In addition, the morphology of PC3 cells grown in ODM changed (***Fig. 1***).

### PC3 cell migration and invasion abilities were enhanced in ODM

**Fig.2:**
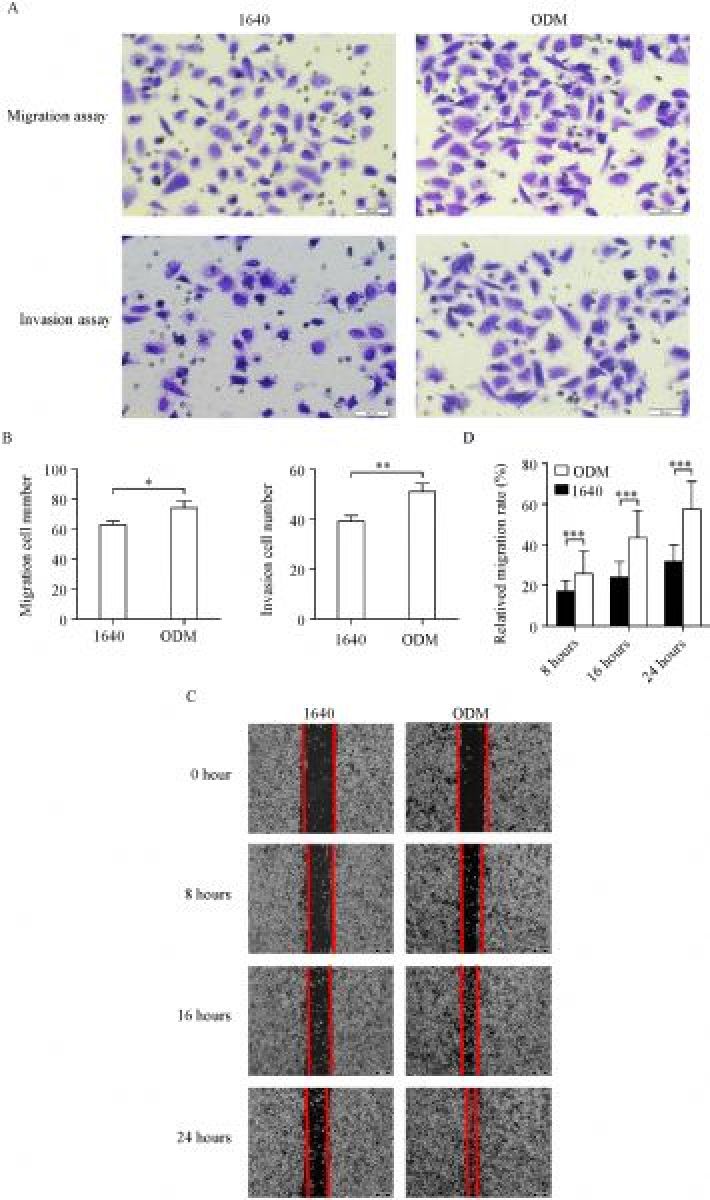
ODM enhances PC3 migration and invasion ***in vitro***.

To assess the abilities of tumor cell migration and invasion *in vitro*, we cultured PC3 cells in 1640 and ODM for two weeks and then subjected the cells to wound-healing and transwell assays. The results showed that cells grown in ODM had significantly enhanced migration (***Fig. 2A ***& ***B***) and invasion abilities (***Fig. 2C ***& ***D***) compared with those grown in 1640.

### NF-κB activation was involved in the alteration of biological behaviors

**Fig.3:**
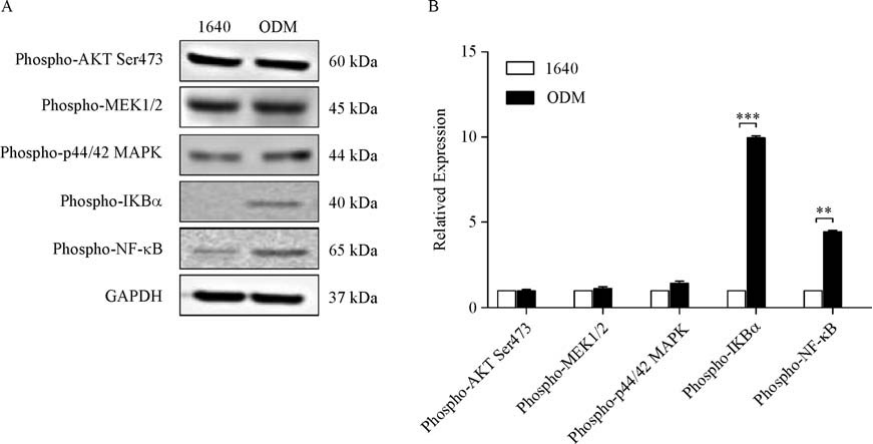
Signaling pathways in PC3 cells grown in bone-mimicking environment.

To identify the signal pathways involved in the alteration of biological behaviors, we detected AKT, MAPK, ERK, and NF-κB pathways, which are related to tumor cell EMT and metastasis, by western blotting. The results showed that NF-κB signaling was activated in the tumor cells grown in ODM (***Fig. 3***). The NF-κB pathway was activated through the phosphorylation of IκBα at Ser32 followed by proteasome-mediated degradation, which resulted in the release and nuclear translocation of active NF-κB. It was indicated that the NF-κB pathway might play an important role in PC3 cells behavior in the bone-mimicking microenvironment.

### NF-κB activation was attenuated dose-dependently by Bay 11-7082

**Fig.4:**
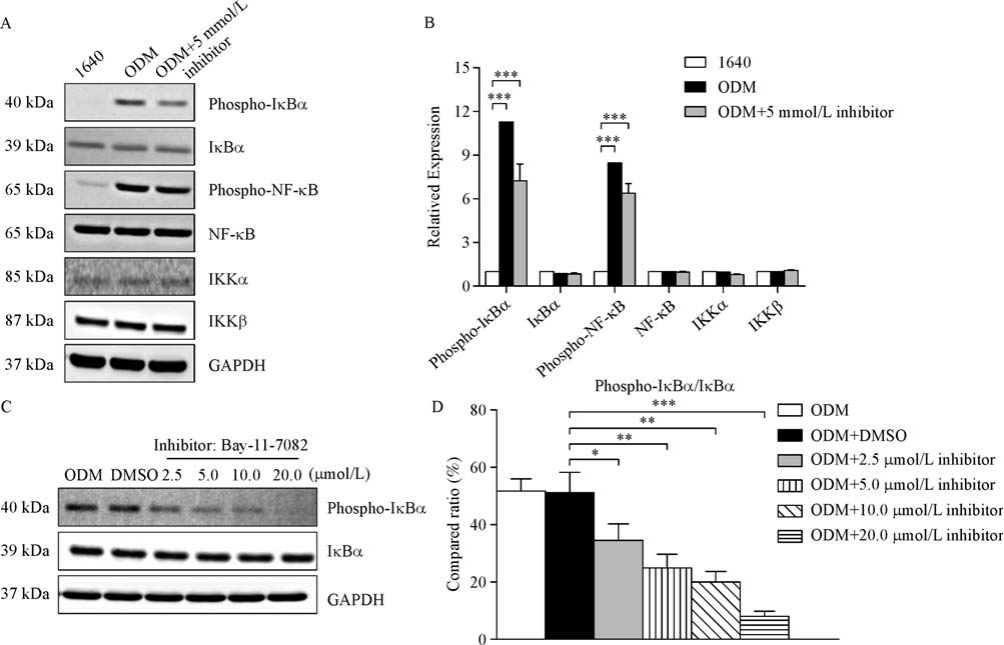
NF-κB signaling activation is attenuated by the NF-κB inhibitor Bay 11-7082.

Bay 11-7082 specifically inhibits NF-κB activation through reducing phosphorylated IκBα in a dose-dependent manner. It is known that NF-κB is sequestered by the inhibitory IκB subunit in the cytoplasm in an inactive form^[24]^. The phosphorylation and degradation of IκBα is essential for the release of active NF-κB. In particular, the phosphorylated site is an excellent marker for NF-κB pathway activation. Bay 11-7082 has an anti-inflammatory effect and contributes to cell apoptosis by interfering with IκBα protein^[25]^. In our study, we found that targeting the NF-κB pathway by Bay 11-7082 reduced key protein expression (***Fig. 4A ***& ***B***). Then, we treated the PC3 cells grown in ODM for 2 hours and examined the effect of Bay 11-7082 (IC50= 10 μmol/L) on NF-κB pathway activity. At 0 to 2.5 μmol/L, Bay 11-7082 did not significantly affect IκBα phosphorylation, whereas it reduced IκBα phosphorylation as of 5.0 μmol/L in a concentration-dependent manner (***Fig. 4C ***& ***D***). With the elevating concentration, Bay 11- 7082 increasingly triggered cell death, as revealed by microscopy. Therefore, we used 5 μmol/L Bay 11-7082 in the subsequent assays to evaluate its effects.

### Bay 11-7082 reversed the biological behaviors of PC3 cells in ODM

**Fig.5:**
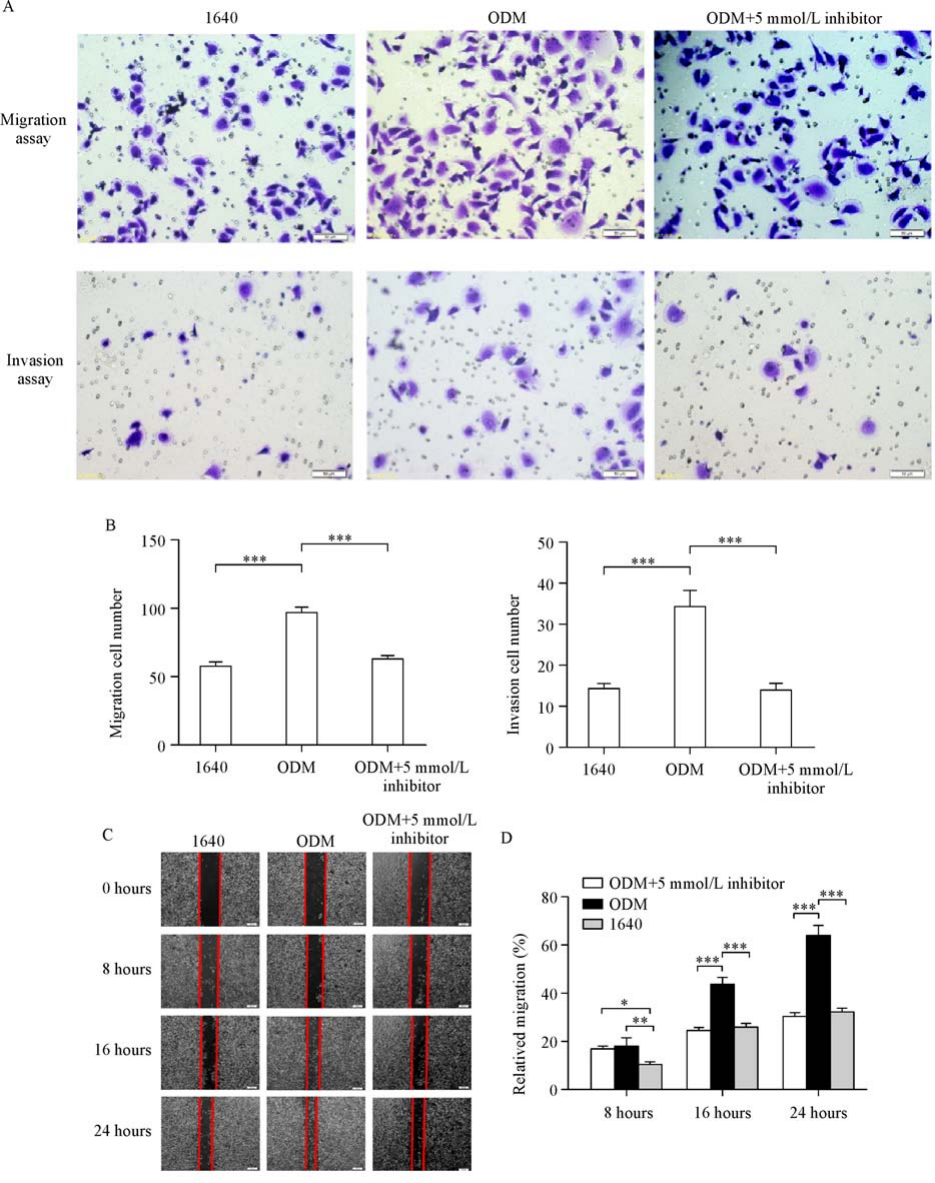
Bay 11-7082 inhibits migration and invasion of PC3 cells grown in ODM.

**Fig.6:**
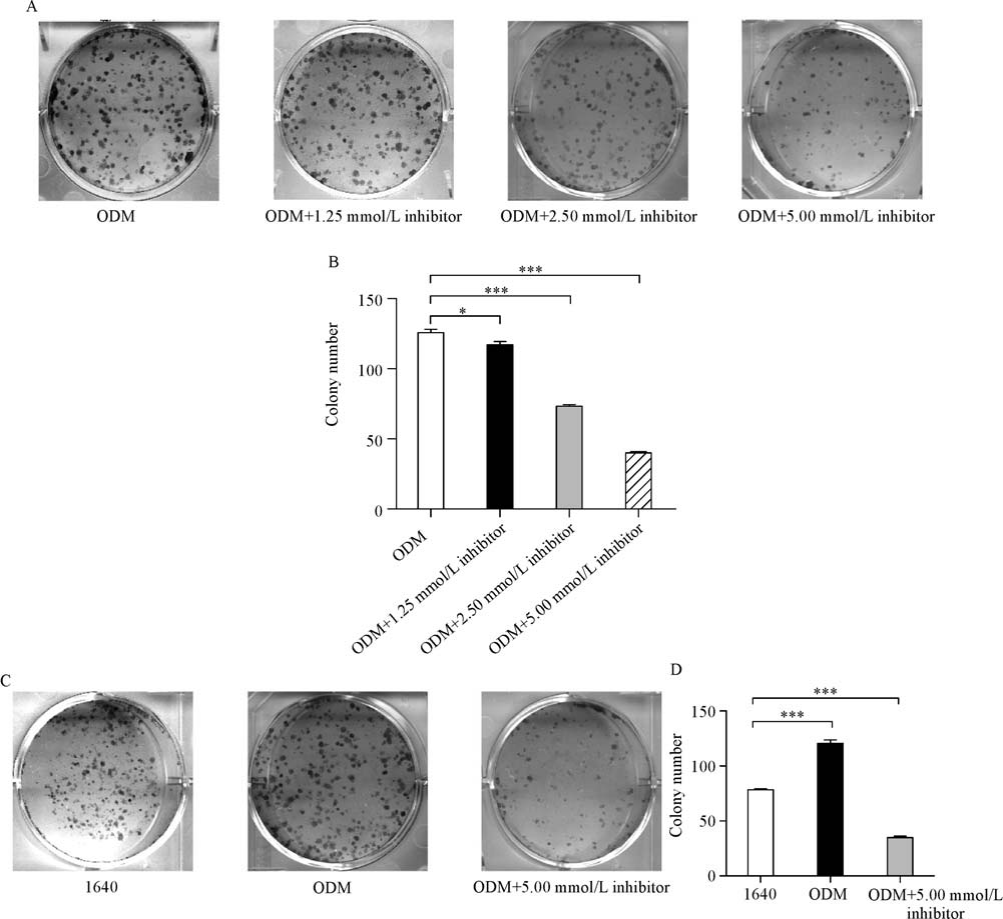
Bay 11-7082 suppresses PC3 cell colony formation in a dose-independent manner.

To determine the effect of Bay 11-7082 on the biological behaviors of PC3 cells grown in ODM, migration, invasion and colony formation assays were performed. We found that through NF-κB pathway activation, ODM induced PC3 cell migration and invasion, which was suppressed by the addition of Bay 11-7082 into the ODM (***Fig. 5***). Further, we observed that ODM enhanced the colony formation ability (CFA), whereas Bay 11-7082 reduced the CFA of PC3 cells in ODM (***Fig. 6***). The numbers of cell clusters were obviously increased in ODM, while Bay 11-7082 inhibited this increase in a dose-dependent way (***Fig. 6***). The results indicated that NF-κB pathway may be associated with the CFA of PC3 cells.

### ODM promoted PC3 cell growth and metastasis in mice

**Fig.7:**
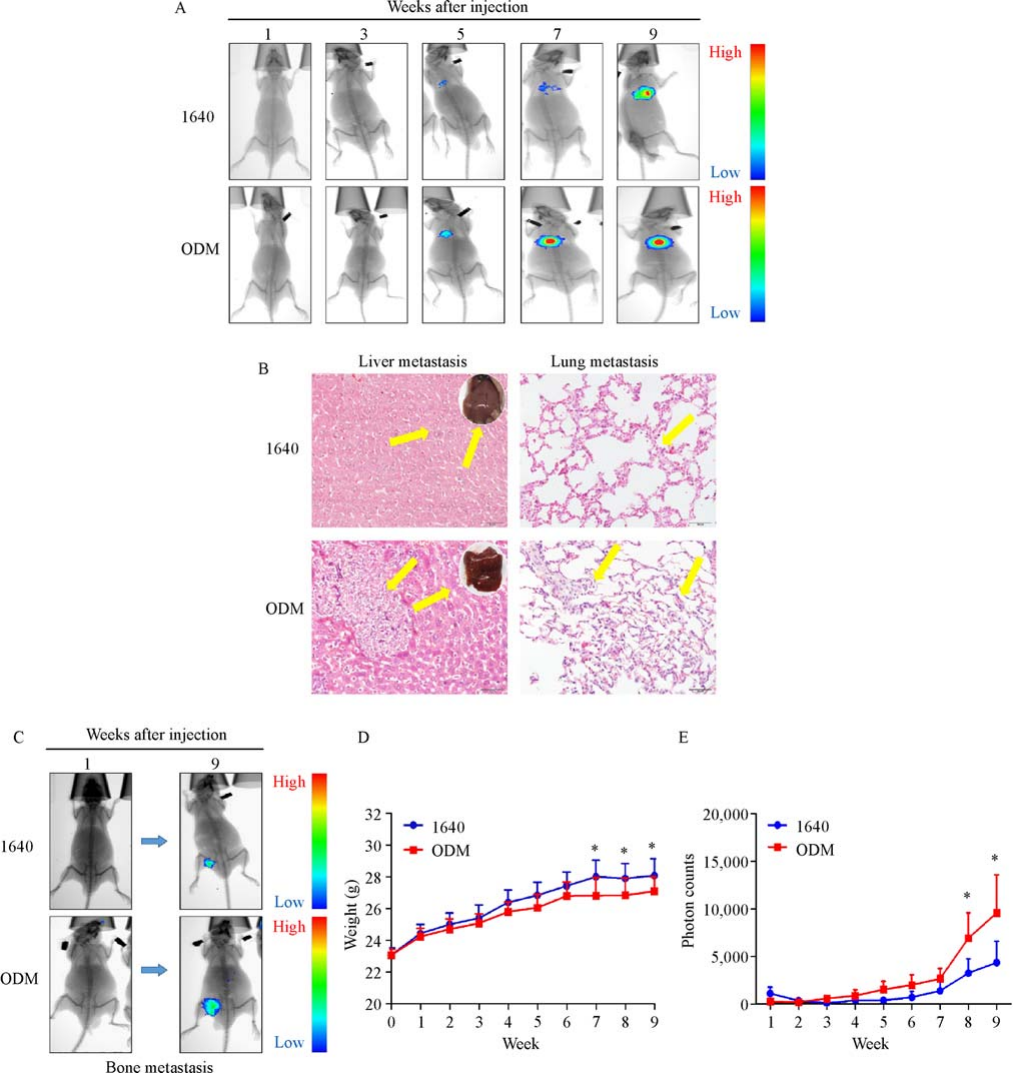
ODM promotes tumor growth and metastasis ***invivo***.

We injected PC3 cells cultured with ODM or 1640 into the left ventricles of immunodeficient nude mice to assess tumor cell growth and metastasis *in vivo*. Tumor dynamic changes were monitored by weekly-captured tumor images (***Fig. 7A ***& ***C***). Ten weeks after injection, the mice were sacrificed, and their livers and lungs were collected and evaluated by HE staining to detect metastasis (***Fig. 7B***). Body weight and tumor growth were recorded once a week (***Fig. 7D ***&*** E***). In the ODM group, 12/13 (92%) mice showed distant organ metastasis, while in the 1640 group only 8/13 (62%) mice did. The mice injected with cells grown in ODM had higher metastatic rates, and tumor cells metastasized to multiple distant organs, such as liver, lung, and bone (***Table 1***).

**Tab.1:** PC3 cells in ODM promoted metastasis to multiple distant organs in mice [***n***(%)]

Group	Liver	Lung	Bone	Total
1640 group (*n*=13)	5 (38)	2 (15)	1 (8)	8 (62%)
ODM group (*n*=13)	7 (54)	3 (23)	2 (15)	12 (92%)

## Discussion

The NF-κB pathway plays pivotal roles in biological processes such as inflammatory immune response, tumor cell proliferation, migration, invasion, and metastasis^[18, 26]^. In the current study, we observed NF-κB pathway activation in PC3 cells cultured in ODM. Interestingly, when PC3 cells were cultured in ODM, their biological behavior changed significantly; they underwent EMT, and showed enhanced migration and invasion abilities. Then we found these processes were significantly delayed or blocked by the NF-κB inhibitor—Bay 11-7082. Further, we observed that ODM promoted PC3 cell growth and metastasis in mice.

Several important molecules and signaling pathways contribute to PCa cell growth and metastasis. In the early stage of EMT, the adhesion molecules (such as E-cadherin and β-catenin) are downregulated and the N-cadherin and vimentin protein levels are upregulated; thus, tumor cells lose their epithelial features and acquire a mesenchymal phenotype, which leads to invasive and migratory behaviors^[27–32]^. Increasing evidence shows that EMT is involved in the development of drug resistance, tumor metastasis^[31]^, and anti-apoptosis^[33–34]^. It is reported that NF-κB signaling is associated with the EMT regulation^[35-36]^. Moreover, NF-κB plays an important role in cancer initiation and progression^[24]^. In this study, after screening several signals, including AKT, MAPK, MEK, and NF-κB, we found that the NF-κB signal pathway was activated, as indicated by the upregulation NF-κB and IκBα phosphorylation. We demonstrated that the activation of the NF-κB pathway promoted EMT, whereas this process was reversed by Bay 11-7082, the NF-κB pathway inhibitor. These results may contribute to a better understanding of NF-κB as a therapeutic target for PCa treatment. It is also confirmed that NF-κB activation drives EMT, and NF-κB regulation might guide PCa cell migration and invasion *in vitro* as well as tumor growth *in vivo*. Furthermore, modulating NF-κB signaling appears to be important since Bay 11-7082 can significantly weaken the migration and invasion abilities of PCa cells and especially, inhibit IκBα phosphorylation and degradation. Therefore, NF-κB is a potential therapeutic against PCa metastasis and our findings warrant further pre-clinical testing of this inhibitor.

Taken together, our findings provide new insights into the mechanism underlying biological behaviors of PC3 cells cultured in different media. The metastasis-related NF-κB signaling pathway is a promising therapeutic target for PCa metastasis.
